# Therapeutic potential of canagliflozin in DEN/TAA-induced renal cancer: mechanistic insights into NLRP3/IL-6/STAT3 and AMPK signaling and oxidative stress regulation

**DOI:** 10.1007/s44446-025-00039-x

**Published:** 2025-10-09

**Authors:** Rehab F. Abdel-Rahman, Mahdi H. Alsugoor, Naif ALSuhaymi, Hany M. Fayed, Sawsan S. Mahmoud, Fatma A. Ibrahim, Marawan A. Elbaset

**Affiliations:** 1 Department of Pharmacology, Medical Research and Clinical Studies Institute, National Research Centre, Giza, 12622 Egypt; 2 Department of Emergency Medical Services, Faculty of Health Sciences, AlQunfudah, Umm Al-Qura University, 21912 Makkah, Saudi Arabia; 3 Biochemistry Department, Biotechnology Research Institute, National Research Centre, Giza, Egypt; 4 Department of Neurology, Indiana University School of Medicine Indianapolis, Indianapolis, IN USA; 5 Stark Neurosciences Research Institute, Indiana University School of Medicine, Indianapolis, IN USA

**Keywords:** Diethyl nitrosamine, Oxidative stress, Thioacetamide, Choline-deficient diet, Renal carcinogenesis, Canagliflozin, Rats

## Abstract

A novel class of antidiabetic drugs known as sodium-glucose cotransporter 2 inhibitors (SGLT2-Is) prevents the renal proximal tubules from reabsorbing glucose. While a recent study showed that SGLT2-Is may be able to slow the proliferation of cancer cells that express SGLT2, limited evidence exists regarding their effects on renal cell carcinoma (RCC). Here, we examine the ability of the SGLT2-I canagliflozin (Cana) to prevent experimentally induced kidney carcinogenesis in male rats. A total of twenty-four rats were divided into four groups, six in each: negative control, DEN/TAA control; rats (60–70 g) were fed a choline-deficient diet (CDD) for 4 weeks, then rats were subjected to four doses of 50 mg/kg diethyl nitrosamine (DEN) over 8 weeks followed by thioacetamide 100 mg/kg (TAA) intraperitoneal injections twice weekly for 15 weeks, treated groups: rats were given canagliflozin (10 and 20 mg/kg b.wt.) orally starting from the 24th week of the experiment till the end of the 29th week. The obtained findings showed that treatment with canagliflozin reduced renal oxidative stress and toxicity indicator levels and considerably reinforced renal antioxidant capacity. The histological changes further supported the biochemical findings. In addition, canagliflozin therapy activated AMPK and inhibited Nrf2, NLRP3 and IL-6/STAT3 pro-inflammatory pathway. Immunohistochemistry exhibited upregulation of pro-apoptotic protein caspase-3 and downregulation of PCNA expression in Cana-treated groups. Conclusion: the results showed that canagliflozin has anti-carcinogenic efficacy against renal carcinogenesis via activating AMPK and suppressing NLRP3/IL-6/STAT3 signaling pathways.

## Introduction

Globally, kidney cancer is among the most prevalent malignant tumors of the genitourinary system. It is the most resistant cancer since conventional treatments have little to no effect on this cancer (Siegel et al. 2019; Xie et al. 2024). Over 400,000 persons are affected with renal cell carcinoma (RCC) annually, making it the third most common urologic cancer (El-Sonbaty et al. 2023). Although partial nephrectomy remains the most successful treatment for localized disease, there is still no appropriate treatment for metastatic cancer because of adverse effects and resistance to chemotherapy and radiation (Hutson et al. 2011; Weiss et al. 2018**)**. Moreover, renal cell carcinoma (RCC) remains a clinically challenging malignancy, with limited curative options and treatment-related toxicities that often compromise long-term outcomes. Thus, there is an urgent need for new, effective therapeutic strategies (Aggarwal et al. 2020).

Increasing evidence supports a pivotal role of inflammation in the initiation and progression of various malignancies, including RCC, through its impact on the tumor microenvironment and immune modulation (Mantovani et al. 2019). The crucial roles that inflammasomes play in the inflammatory process have been well investigated (von Moltke et al. 2013). The most well-known is the NLRP3 (nucleotide-binding domain, leucine-rich–containing family, pyrin domain–containing-3) inflammasome (Strowig et al. 2012). Cytokines like “tumor necrosis factor (TNF-α), IL-1 beta (IL-1β), and interleukin-6 (IL-6)” are released into the tumor microenvironment by the inflammasome from necrotic cells (Hanahan and Weinberg 2011). According to recent research, NLRP3 is crucial for the development of cancer, including lung cancer (Kong et al. 2015), bladder cancer (Poli et al. 2015) and acute lymphoblastic leukemia (Paugh et al. 2015). NLRP3 expression was shown to be increased in RCC (Wang et al. 2019). Additionally, signal transducer and activator of transcription 3 (STAT3) plays a crucial role in the beginning and progression of RCC and is necessary for angiogenesis, apoptosis, and survival (Lorente et al. 2019). STAT3 activation promotes tumor angiogenesis in a variety of tumor types by upregulating the production of vascular endothelial growth factor (VEGF) and metalloproteinases (MMPs) (Santoni et al. 2013). The cytokine IL-6 is primarily responsible for activating Janus kinases (JAK)/STAT3 signaling (Kamimura et al. 2003). JAK enzymes are triggered when IL-6 attaches to its receptor, phosphorylate STAT3. When STAT3 is phosphorylated, it enters the nucleus and alters the transcription of genes that produce inflammatory factors (Li et al. 2019). The anti-inflammatory qualities of canagliflozin were confirmed in prior studies (Xu et al. 2018). Thus, we hypothesized that canagliflozin might prevent renal cancer from progressing by regressing NLRP3/IL-6/STAT3 activity.

An indication of an energy deficit, the AMP-activated protein kinase (AMPK) is a cellular energy sensor that undergoes phosphorylation and increases activity in response to an increase in AMP and a drop in ATP (Hardie 2011). Notably, it has been demonstrated that AMPK activation lowers inflammation and improves insulin sensitivity (Ge et al. 2022). AMPK activation has been shown in numerous studies to suppress the mitogen-activated protein kinase (MAPK) and nuclear factor-κB p65 (NF-κB p65) signaling pathways (Wu et al. 2023). Furthermore, the JAK1/STAT3 pathway's proteins are directly phosphorylated by AMPK, which inhibits this pathway (Fonseca et al. 2022). AMPK was first identified as a tumor suppressor (Liang and Mills 2013). AMPK activators have strong inhibitory effects on RCC cells, and pharmacological targeting of AMPK may offer a unique therapy strategy for a number of cancers (Woodard et al. 2010). As a result, AMPK activation is viewed as a potential strategy by canagliflozin to mitigate the reno-carcinogenesis caused by DEN/TAA.

The overexpression of sodium-glucose cotransporter 2 (SGLT2), a crucial glucose transporter in cancer models, has been connected to increased glucose uptake in humans and animals. By suppressing its expression, tumor growth can be successfully prevented both in vitro and in vivo (Basak et al. 2023). The novel SGLT2 inhibitor canagliflozin can lower renal glucose reabsorption and decrease patients’ glycemic levels (Chao 2014). Preclinical studies showed that canagliflozin not only regulated blood sugar levels but also reduced the development of prostate, pancreatic, and lung cancer (Villani et al. 2016). Canagliflozin has been shown to have anti-inflammatory and antioxidant qualities, lowering blood pressure, heart and kidney remodeling, and arterial stiffness (Hasan et al. 2020). According to recent research, SGLT2 inhibitors reduce inflammation by specifically targeting the NLRP3 inflammasome (Pawlos et al. 2021). According El-Dessouki et al. study (El-Dessouki et al. 2025), pretreatment with canagliflozin reduced the expression of the JAK1/STAT3 protein in methotrexate-induced liver damage, indicating their anti-inflammatory qualities. According to Hasan et al. study (2020), Canagliflozin therapy inhibited pro-oxidative/pro-inflammatory and pro-apoptotic signaling via caspase-3 activation while stimulating antioxidant/anti-inflammatory signaling pathways involving AMPK. It resulted in a notable increase in kidney function in rats with renal oxidative injury caused by isoprenaline.

Given the multifaceted pharmacological potential of canagliflozin and its reported anti-inflammatory, antioxidant, and metabolic effects, this study was designed to evaluate its chemopreventive efficacy in a diethylnitrosamine (DEN)/thioacetamide (TAA)-induced model of renal carcinogenesis in rats. We further aimed to elucidate the underlying molecular pathways, with a particular focus on NLRP3/IL-6/STAT3 and AMPK signaling axes.

## Materials and methods

### Animals

For this study, one-month-old weaned rats weighing 50 and 80 g were chosen and obtained from the National Research Centre's (NRC) animal house colony in Giza, Egypt. Rats were housed in cages that were 25 ± 0.5 °C, had proper ventilation, sixty percent humidity, and with equal light–dark cycles, without enrichments. All groups of animals, with the exception of the negative control, were housed in hygienic cages and fed a choline-deficient diet (CDD) for four weeks. This was followed by a standard pellet diet until the end of the experiment, with water provided ad libitum. The experimental protocol was licensed (CU23052022441) by “Cairo University Institutional Animal Care and Use Committee” (IACUC).

### Drugs and chemicals

TAA (Lot#BCBT1576) and DEN (Lot#MKCK720) were obtained from Sigma-Aldrich (Germany). Canagliflozin tablets (Invokana®) were acquired from “Nile Pharmaceutical Company in Egypt”. Tablets were crushed into a fine powder and freshly suspended in sterile saline immediately before oral administration by gavage.

### Reno-carcinogenesis model

**Fig. 1:**
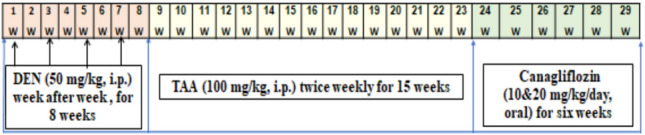
Diagrammatic illustration of the experimental design

As depicted in Fig. 1, rats received four intraperitoneal DEN (50 mg/kg) injections, each injection week after week (for 8 weeks) (Kurma et al. 2021). Subsequently, animals were injected intraperitoneally with TAA at 100 mg/kg dosage and administered twice weekly for 15 weeks (Ayoub et al. 2022). Daily oral treatment with canagliflozin for 6 successive weeks started after 23 weeks of DEN/TAA injections, as represented in Fig. 1.

### Experimental design

At random, twenty-four rats were split up into four groups of six rats in each group:

Group-I (negative control): the rats received the vehicle (saline) once daily, intraperitoneally (ip), and all over the experimental period.

Group-II (DEN/TAA control): received DEN/TAA until the 24th week, after which it was discontinued, and a comparable vehicle was provided without DEN/TAA till the end of the study. Group-III and IV: (treated groups): kidney cancer group rats were orally administered canagliflozin at doses of (10 and 20 mg/kg/day for 6 weeks) (Mabrouk Gabr and Hassan Mohammed 2020) starting from the 24th week till the end of the experiment.

### Sacrification and biological sample collection

The rats underwent anesthesia to acquire blood samples from the tail- vein using light phenobarbital. Sera were utilized right away for the biochemical analysis after being separated by centrifugation. Kidneys were removed, washed with saline, blot-dried, and split into three sections; part was immersed in 10% buffered formalin for histopathological and immunohistochemical analysis. Another portion was homogenized in 10% wt/vol phosphate-buffered saline, and the resulting supernatant was utilized for the ELISA assay and to evaluate oxidative stress biomarkers. The remaining part was frozen at −80°C for PCR analysis.

### Kidney injury indicators

The kits from the Biodiagnostic Company (Cat# UR 21 10 and CR 12 51, respectively) were used to measure serum urea and creatinine levels.

### Renal oxidation stress markers

The renal levels of reduced glutathione (GSH) (Cat# GR2511), superoxide dismutase (SOD) (Cat# SD2521), and malondialdehyde (MDA) (Cat# MD2529) were measured using kits obtained from “Bio-diagnostic, Egypt” in accordance with the manufacturer’s instructions.

### Inflammatory markers

Renal STAT3 (Cat# SL1672Ra) and p-STAT3 (Cat# SLD1757Ra) levels were determined with the ELISA technique using Sunlong Biotech Co., Ltd., China kits.

### Measurement of AMPK, and Nrf2 levels

Using the ELISA technique, the levels of NF-E2-related factor 2 (Nrf2) (Cat# SL0985Ra), AMP-activated protein kinase (AMPK) (Cat# SL1695Ra), and p-AMPK (Cat# SL0570Ra) in kidney tissues were measured, following the manufacturer’s protocol using Sunlong Biotech Co., Ltd., China kits.

### Quantitative real-time PCR to measure IL-6 and NLRP3 gene expression

After adding 30 mg of the tissue sample to 600 µl of RLT buffer that contained 10 μl β mercaptoethanol per 1 ml, the “QIAamp RNeasy Mini kit (Qiagen, Germany, GmbH)” was used to extract RNA from the tissue samples. Tubes were inserted into adapter sets, which are secured to the Qiagen tissue Lyser clamps to homogenize the samples. A two-minute high-speed (30 Hz) shaking step was used to cause disruption. Following the Purification of Total RNA from Animal Tissue's methodology of the “QIAamp RNeasy Mini kit (Qiagen, Germany, GmbH)”, a volume of 70% ethanol was added to the cleared lysate. Note: DNase digestion was performed on the column to eliminate any remaining DNA. The Oligonucleotide primers used are indicated in Table 1 and were provided by Metabion (Germany). A 25 µl reaction comprising 12.5 µl of the 2 × QuantiTect SYBR Green PCR Master Mix (Qiagen, Germany, GmbH), 0.25 µl of RevertAid Reverse Transcriptase (200 U/µL) (Thermo Fisher), 0.5 µl of each primer at a concentration of 20 pmol, 8.25 µl of water, and 3 µl of RNA template was used to use the primers. A Stratagene MX3005P real-time PCR equipment was used to conduct the reaction. The Stratagene MX3005P program was used to determine amplification curves and Ct values. In accordance with Yuan et al. (2006) “ΔΔCt” approach, the Ct of each sample was compared with that of the DEN/TAA control group using the following ratio: (2-DDct) in order to assess the variation of gene expression on the RNA of the various samples. However, ΔΔCt is equal to “ΔCt reference—ΔCt target”. “ΔCt target = Ct control –Ct treatment and ΔCt reference = Ct control- Ct treatment”.

**Table 1:** A list of primers for qPCR

“Target genes”		“Primers sequences”	Amplified fragment length
**IL-6 (**Begue et al. 2013**)**	F	TGTATGAACAGCGATGATG	128 bp
	R	AGAAGACCAGAGCAGATT	
**NLRP3 (**Samra et al. 2016**)**	F	CAGACCTCCAAGACCACGACTG	127 bp
	R	CATCCGCAGCCAATGAACAGAG	
**Rat ß. Actin (**Banni et al. 2010**)**	F	TCCTCCTGAGCGCAAGTACTCT	153 bp
	R	GCTCAGTAACAGTCCGCCTAGAA	

### Histopathological examination and grading of histological changes

Kidney specimens were fixed in 10% neutral buffer formalin, then trimmed, washed in water, dehydrated in ascending grades of ethyl alcohol, cleared in xylene and embedded in paraffin. Thin Sects. (4-6µ) were processed and stained with Hematoxylin & Eosin stain (H & E) **(**Bancroft and Gamble 2008**).** All histological photomicrographs were captured at a magnification of × 400, with a scale bar of 25 μm shown in each image.

### Immunohistochemistry and histological scoring methodology

Immunohistochemical (IHC) staining was performed on paraffin-embedded renal tissue sections using the streptavidin–biotin-peroxidase method (Abdel-Rahman et al. 2019). Antigen retrieval was achieved by heating slides in citrate buffer (10 mM, pH 6.0) for 20 min at 95 °C. Endogenous peroxidase activity was quenched using 0.3% hydrogen peroxide in methanol for 10 min at room temperature, followed by blocking with 5% normal goat serum for 30 min. Sections were incubated overnight at 4 °C with primary antibodies against caspase-3 (rabbit polyclonal antibody, at a dilution of 1:100, A11953; ABclonal, MA, USA) and PCNA (rabbit polyclonal antibody, at a dilution of 1:1000, A0264; ABclonal, MA, USA), then with biotinylated secondary antibody (1:500, 30 min at room temperature) and HRP-conjugated streptavidin. Visualization was achieved using 3,3′-diaminobenzidine (DAB, produced by Sigma) and counterstained with hematoxylin.

Positive controls were tissues known to express caspase-3 and PCNA, demonstrating appropriate localization and intensity. Negative controls included adjacent sections processed without the primary antibody, confirming the specificity of staining and absence of background signal.

Quantification of IHC staining was conducted using ImageJ software by color deconvolution according to (Varghese et al. 2014).

### Statistical analysis

The scientist was blinded during the analysis of the entire study. Before any statistical analysis, normality was checked using the Shapiro test, and heteroscedasticity was checked using the Brown-Forsythe test. We used Tukey's post-hoc test after presenting the data as means ± SD and analyzing it with a one-way ANOVA. In order to ascertain the statistical inference of the data, we simultaneously displayed non-parametric data as median ± interquartile range and employed the Kruskal–Wallis test and Dunn's test. GraphPad Prism software (version 10, CA, San Diego, USA) displayed the differential inference as an asterisk. Every statistical test that has a *P*-value of less than 0.05 was deemed significant.

## Results

### Effect of Canagliflozin on Serum Kidney Function Markers in DEN/TAA-Induced Renal Cancer

**Fig. 2:**
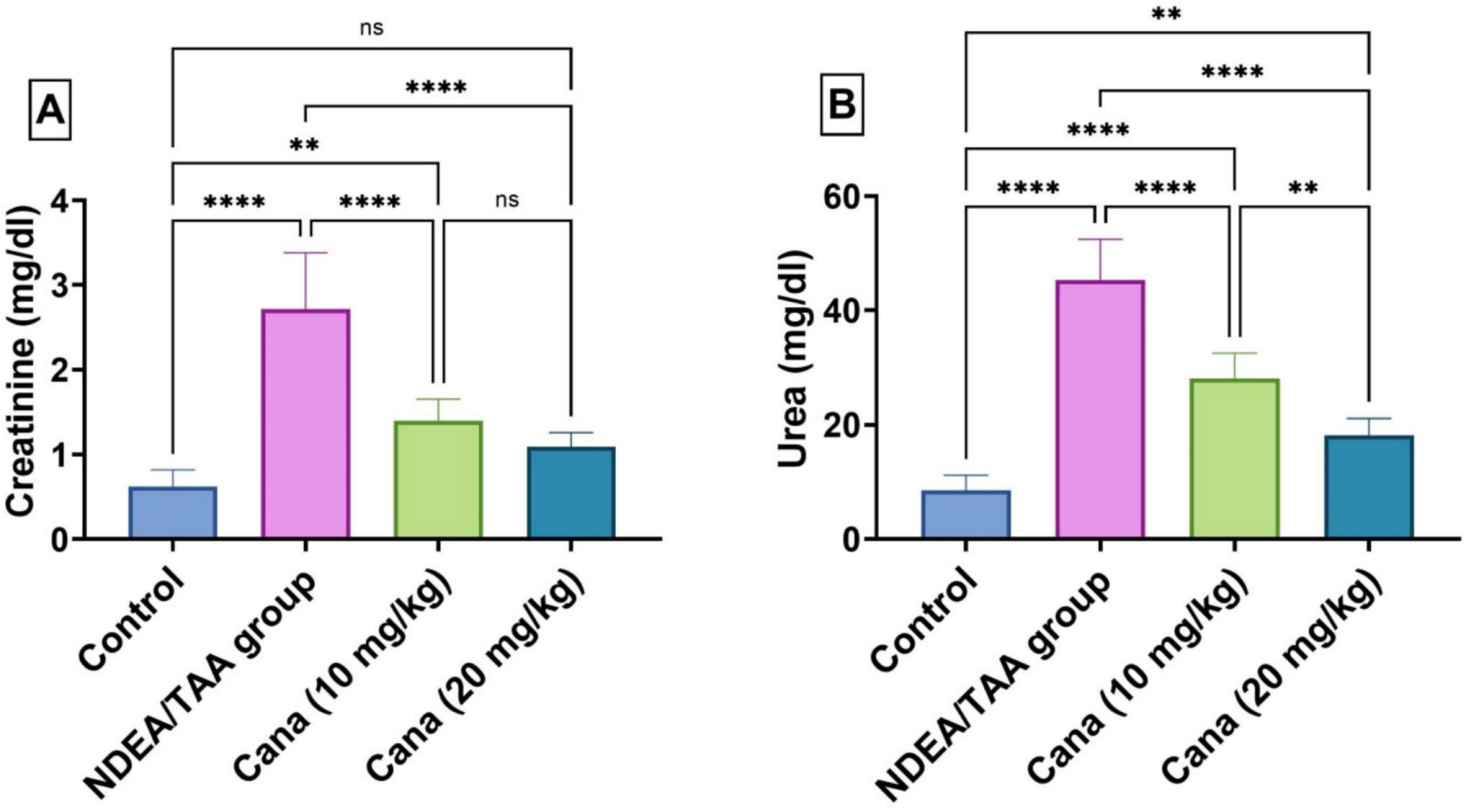
Assessment of canagliflozin effect on kidney function: creatinine and urea in DEN/TAA-Administered rats. (**A**) Serum creatinine (mg/dl). (**B**) Serum urea (mg/dl). Six rats’ mean ± SD was used to present the data, with *p*-values displayed on the bars * (*P* ≤ 0.05), ** (*P* ≤ 0.01), *** (*P* ≤ 0.001), **** (*P* ≤ 0.0001). DEN/TAA: Diethylnitrosamine/Thioacetamide; Cana: Canagliflozin

DEN/TAA administration induced a significant elevation in serum creatinine and urea by 338.71% (1.22 ± 1.313 vs. 0.3 ± 0.285, mg/dl) and 430.18% (18.45 ± 23.373 vs. 4.09 ± 3.94, mg/dl), respectively, compared to the control group. Treatment with canagliflozin showed dose-dependent improvement, where high-dose canagliflozin (HD-Cana) normalized creatinine levels nearly to control values (75.8% improvement from DEN/TAA) (0.44 ± 0.564 vs. 1.22 ± 1.313, mg/dl), while low-dose canagliflozin (LD-Cana) showed moderate improvement (48.5% improvement) (0.59 ± 0.709 vs. 1.22 ± 1.313, mg/dl). Similarly, urea levels were significantly improved with HD-Cana (59.9% improvement) (7.43 ± 9.341 vs. 18.45 ± 23.373, mg/dl) and LD-Cana (38.2% improvement) (11.45 ± 14.392 vs. 18.45 ± 23.373, mg/dl), though not completely normalized to control levels (Fig. 2).

### Effect of Canagliflozin on AMPK signaling in DEN/TAA-induced renal cancer

**Fig. 3:**
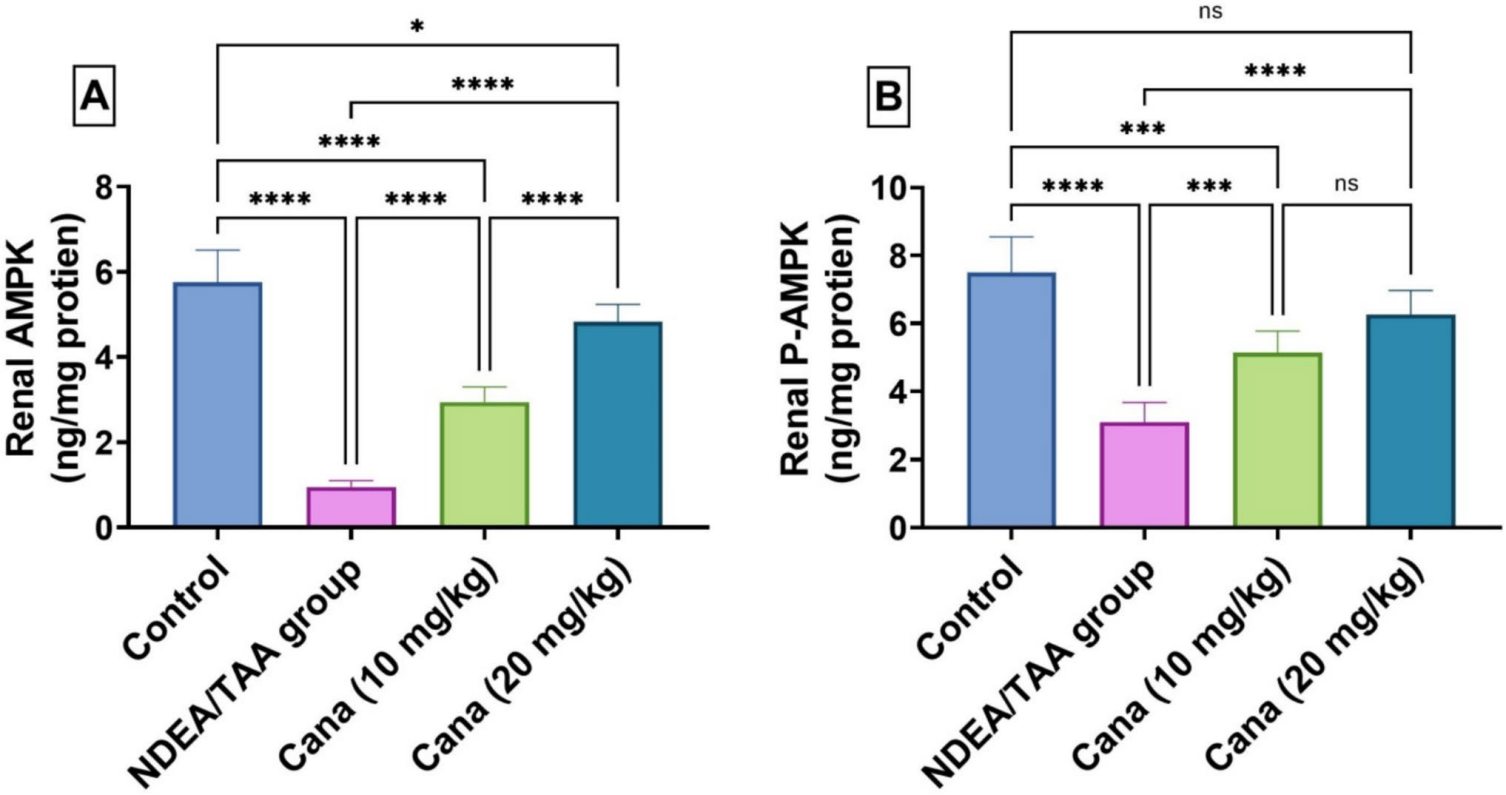
Assessment of Canagliflozin effect on kidney AMPK and P-AMPK in DEN/TAA-administered rats. **(A)** AMPK (ng/mg protein). **(B)** P-AMPK (ng/mg protein). Six rats’ mean ± SD was used to present the data, with *p*-values displayed on the bars * (*P* ≤ 0.05), ** (*P* ≤ 0.01), *** (*P* ≤ 0.001), **** (*P* ≤ 0.0001). DEN/TAA: Diethylnitrosamine/Thioacetamide; Cana: Canagliflozin

DEN/TAA significantly decreased renal AMPK and P-AMPK levels by 83.48% (0.11 ± 0.063 vs. 0.53 ± 0.317, ng/mg protein) and 58.67% (0.41 ± 0.243 vs. 0.74 ± 0.439, ng/mg protein), respectively, compared to control, with the P-AMPK/AMPK ratio increasing from 1.304 to 3.263, indicating disrupted AMPK signalling. HD-Cana treatment significantly restored both AMPK (408% improvement from DEN/TAA) (0.29 ± 0.17 vs. 0.11 ± 0.063, ng/mg protein) and P-AMPK (102% improvement) (0.49 ± 0.293 vs. 0.41 ± 0.243, ng/mg protein), nearly normalizing the P-AMPK/AMPK ratio. LD-Cana showed moderate improvements in both parameters (208% (0.25 ± 0.151 vs. 0.11 ± 0.063, ng/mg protein) and 65.5% 0.45 ± 0.269 vs. 0.41 ± 0.243, ng/mg protein) improvement, respectively), though not reaching control levels (Fig. 3).

### Effect of Canagliflozin on Nrf2 in DEN/TAA-induced renal cancer

**Fig. 4:**
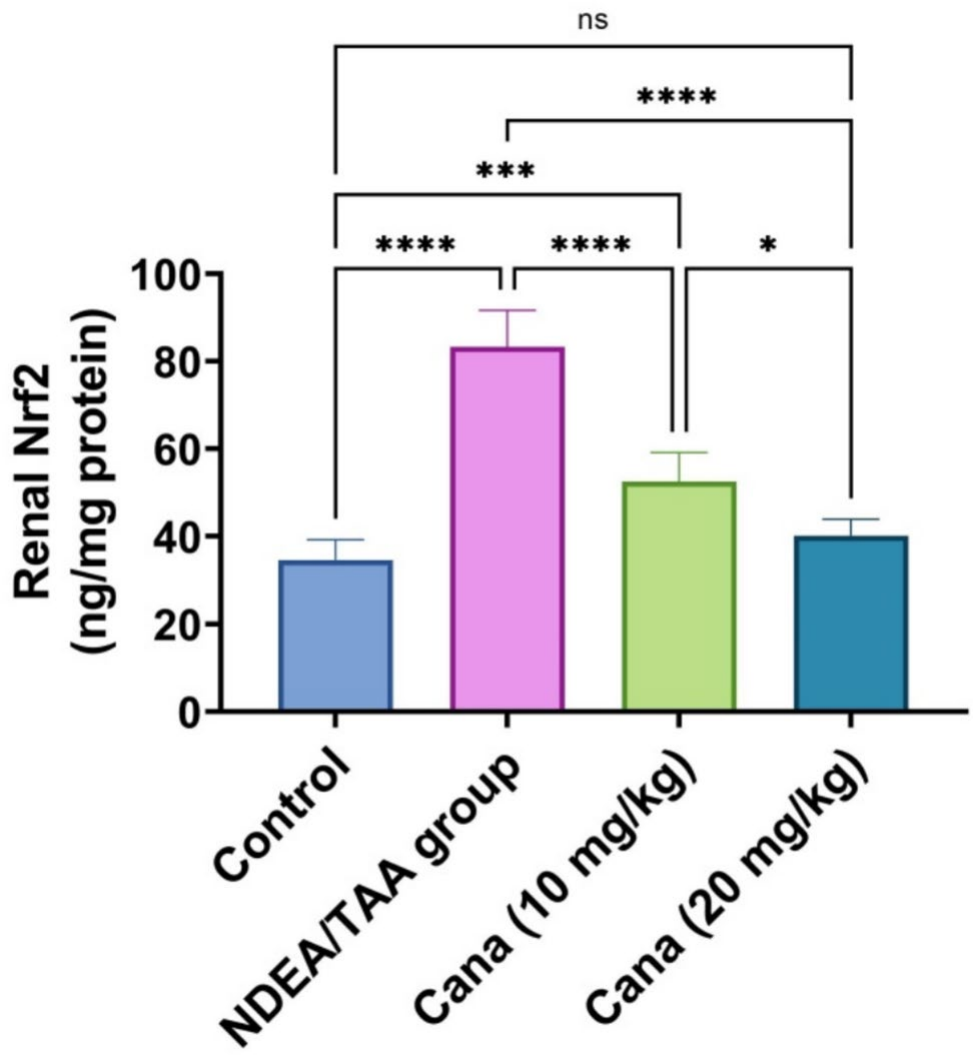
Assessment of Canagliflozin effect on kidney Nrf2 in DEN/TAA-Administered Rats. Six rats’ mean ± SD was used to present the data, with *p*-values displayed on the bars * (*P* ≤ 0.05), ** (*P* ≤ 0.01), *** (*P* ≤ 0.001), **** (*P* ≤ 0.0001). DEN/TAA: Diethylnitrosamine/Thioacetamide; Cana: Canagliflozin

DEN/TAA administration caused a significant increase in Nrf2 (140.35% increase) (31.71 ± 44.772 vs. 13.69 ± 18.218, ng/mg protein) compared to the control. HD-Cana treatment significantly normalized Nrf2 levels (51.8% improvement) (15.13 ± 21.712 vs. 31.71 ± 44.772, ng/mg protein). LD-Cana showed intermediate improvement in Nrf2 (37% improvement) (20.62 ± 27.675 vs. 31.71 ± 44.772, ng/mg protein), though not reaching control levels (Fig. 4).

### Effect of canagliflozin on STAT3 Signaling in DEN/TAA-Induced Renal Cancer

**Fig. 5:**
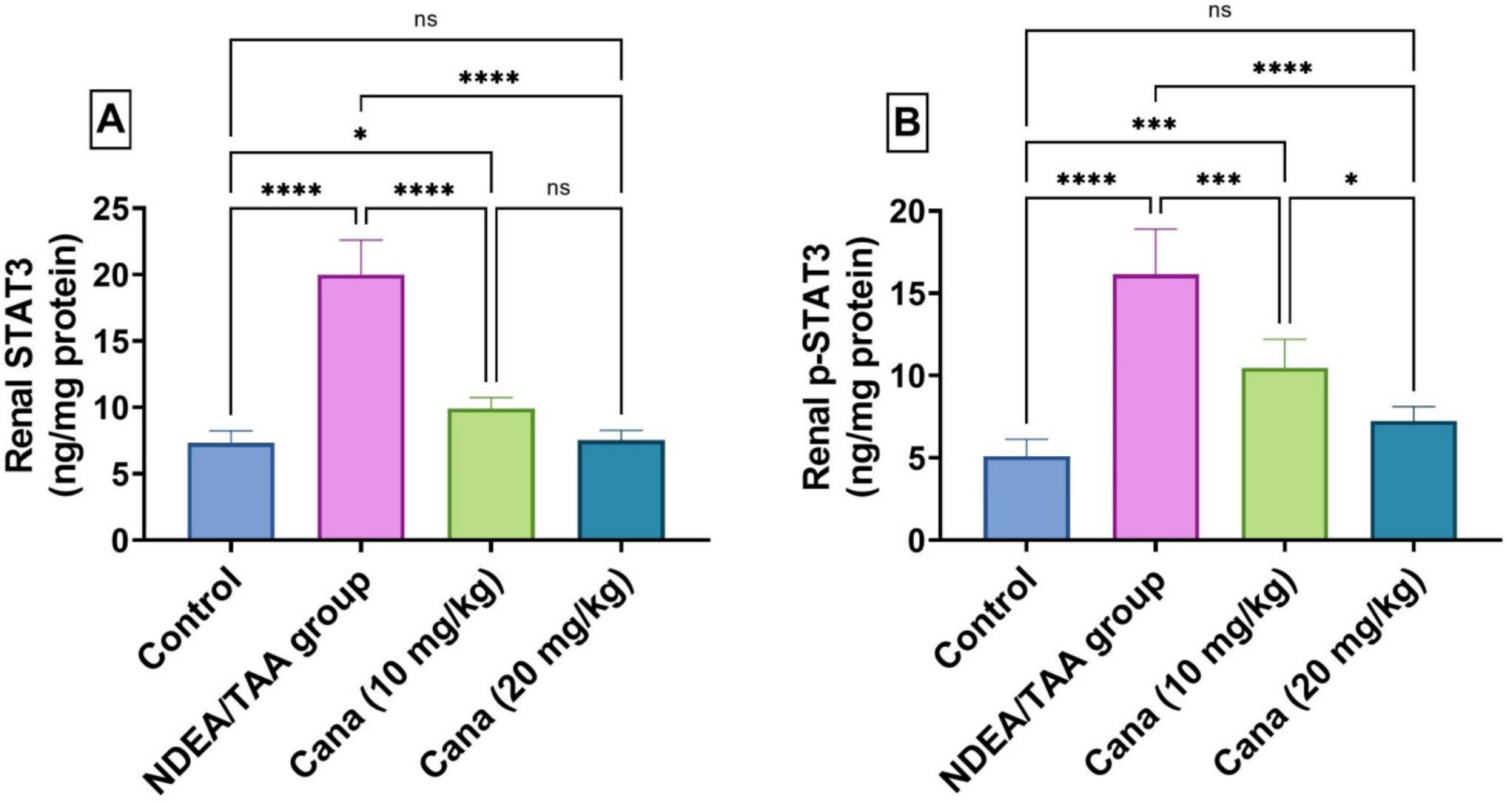
Assessment of Canagliflozin effect on kidney STAT3 and p-STAT3 in DEN/TAA-administered rats. (**A**) STAT3 (ng/mg protein). (**B**) p-STAT3 (ng/mg protein). Six rats’ mean ± SD was used to present the data, with *p*-values displayed on the bars * (*P* ≤ 0.05), ** (*P* ≤ 0.01), *** (*P* ≤ 0.001), **** (*P* ≤ 0.0001). DEN/TAA: Diethylnitrosamine/Thioacetamide; Cana: Canagliflozin

DEN/TAA significantly increased both STAT3 and P-STAT3 levels by 173.22% (7.89 ± 10.515 vs. 2.88 ± 3.855, ng/mg protein) and 216.27% (6.67 ± 8.23 vs. 2.18 ± 2.543, ng/mg protein), respectively, with the P-STAT3/STAT3 ratio shifting from 0.697 to 0.806. HD-Cana treatment effectively normalized both STAT3 and P-STAT3 levels (62.4% (2.86 ± 4.036 vs. 7.89 ± 10.515, ng/mg protein) and 55.2% (2.83 ± 3.826 vs. 6.67 ± 8.23, ng/mg protein) improvement from DEN/TAA, respectively). LD-Cana showed moderate improvement (50.5% (3.7 ± 5.377 vs. 7.89 ± 10.515, ng/mg protein) and 35.1% (4.3 ± 5.365 vs. 6.67 ± 8.23, ng/mg protein) improvement, respectively), though not completely normalizing the P-STAT3/STAT3 ratio (Fig. 5).

### Effect of Canagliflozin on oxidation stress indicators in DEN/TAA-induced renal cancer

**Fig. 6:**
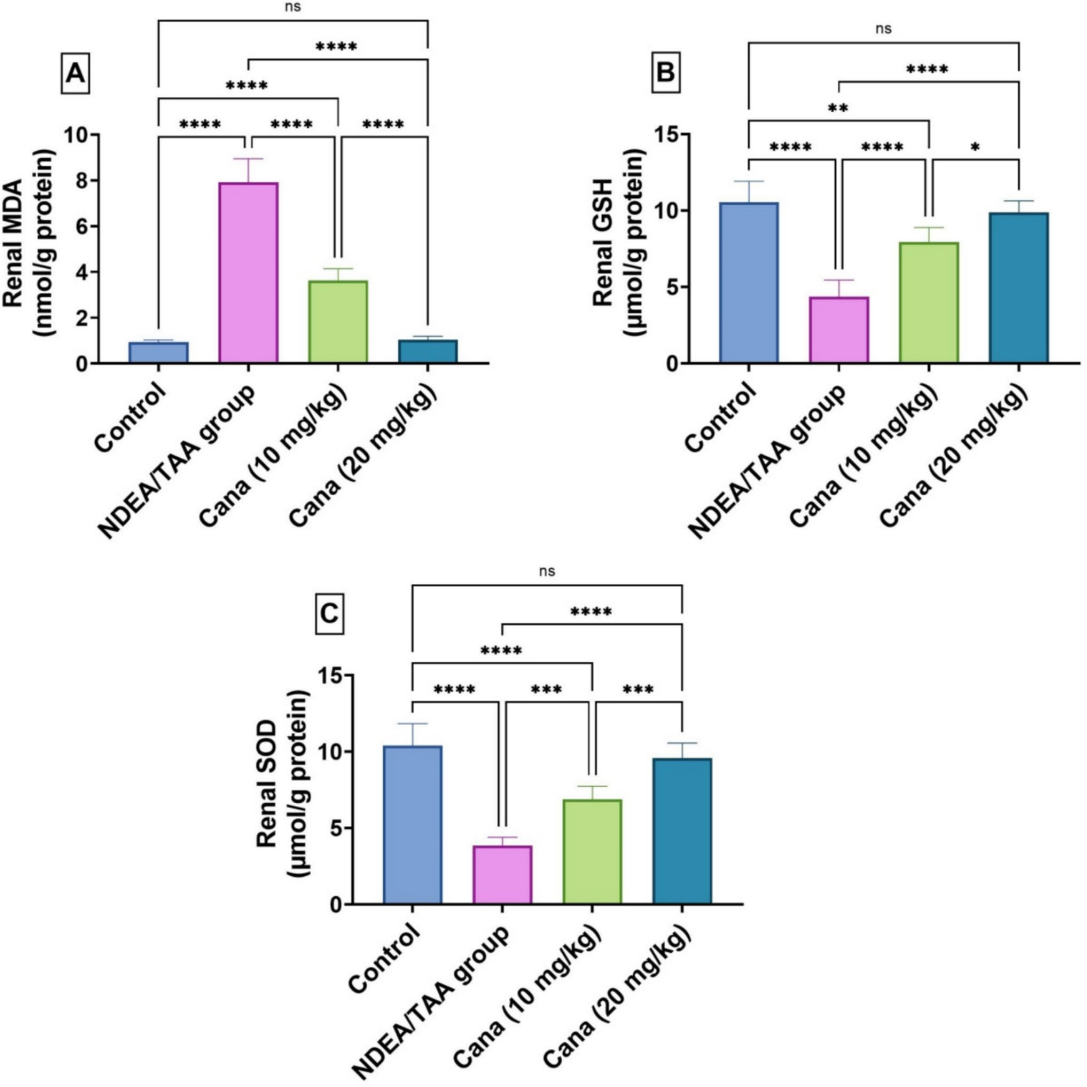
Assessment of canagliflozin effect on oxidative stress markers: MDA, GSH and SOD in DEN/TAA-administered rats. (**a**) MDA (nmol/mg protein), (**b**) GSH (nmol/mg protein), (**c**) SOD (U/mg protein) activity. Six rats’ mean ± SD was used to present the data, with *p*-values displayed on the bars * (*P* ≤ 0.05), ** (*P* ≤ 0.01), *** (*P* ≤ 0.001), **** (*P* ≤ 0.0001). DEN/TAA: Diethylnitrosamine/Thioacetamide; Cana: Canagliflozin

DEN/TAA administration caused severe oxidative stress in liver tissues, demonstrated by a substantial increase in malondialdehyde (MDA) levels by 7.4-fold compared to the control group (3.12 ± 4.165 vs. 0.36 ± 0.502, nmol/g protein), indicating elevated lipid peroxidation. In contrast, antioxidant defence was significantly compromised, with reduced glutathione (GSH) levels decreasing by 58.58% (1.97 ± 2.105 vs. 4.16 ± 5.552, μmol/g protein) and superoxide dismutase (SOD) activity (1.54 ± 2.02 Vs. 4.14 ± 5.452, μmol/g protein) declining by 62.86% relative to controls. Treatment with high-dose canagliflozin (HD-Cana) markedly improved oxidative status by reducing MDA levels by 86.7% (0.42 ± 0.545 vs. 3.12 ± 4.165) from the DEN/TAA group and significantly increasing GSH and SOD levels by 126.1% (3.65 ± 5.406 vs. 1.97 ± 2.105, μmol/g protein) and 147.5% (3.66 ± 5.137 vs. 1.54 ± 2.02, μmol/g protein), respectively, restoring them close to normal control values. Low-dose canagliflozin (LD-Cana) also improved these oxidative stress markers but to a lesser extent, with partial recovery of MDA, GSH, and SOD that did not reach full normalization **(**Fig. 6**)**.

### Effect of Canagliflozin on inflammatory markers in DEN/TAA-induced renal cancer

**Fig. 7:**
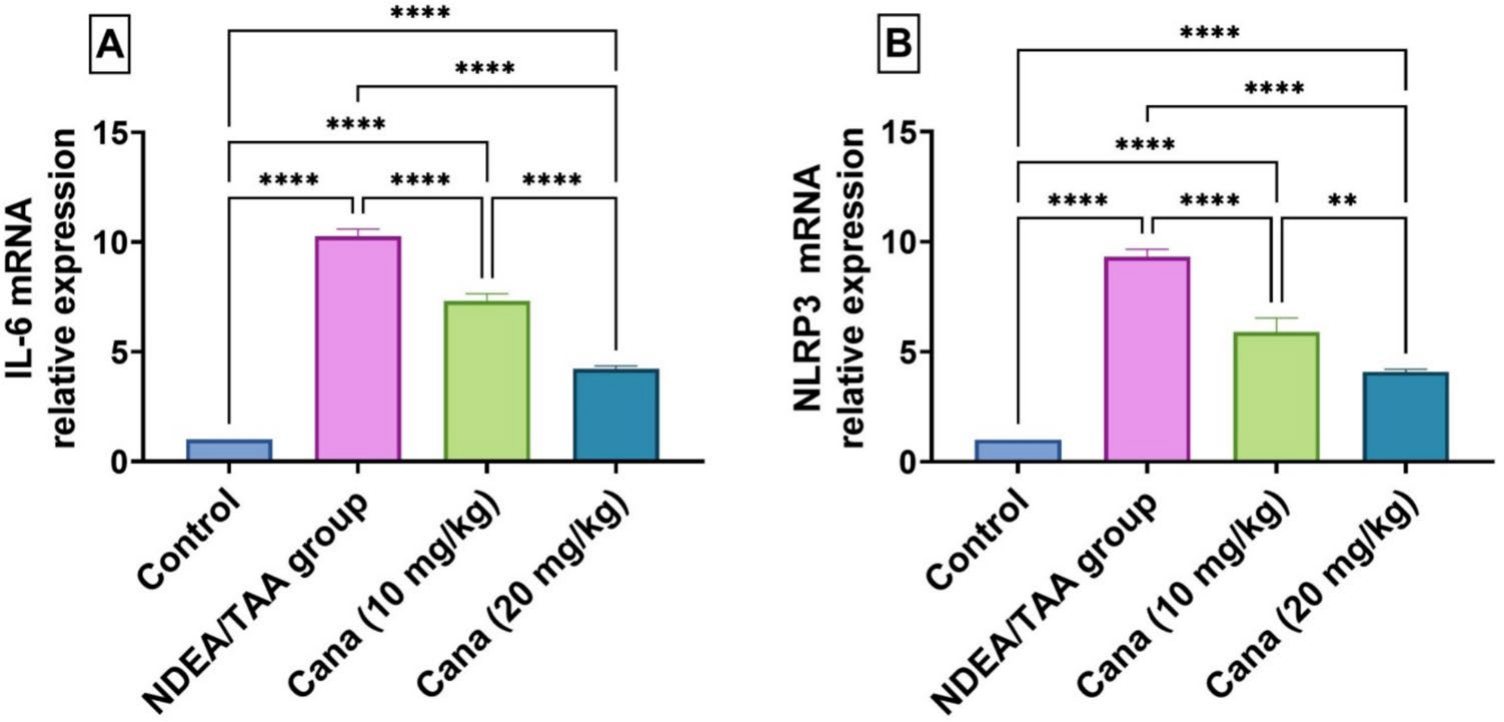
Assessment of canagliflozin effect on Inflammatory Mediators: IL-6 and NLRP3 in DEN/TAA-Administered Rats. (**A**) IL-6 gene expression. (**B**) NLRP3 gene expression. Each bar represents the mean ± SD was used to present the data, with *p*-values displayed on the bars * (*P* ≤ 0.05), ** (*P* ≤ 0.01), *** (*P* ≤ 0.001), **** (*P* ≤ 0.0001). DEN/TAA: diethylnitrosamine/thioacetamide; cana: canagliflozin

As depicted in Fig. 7, DEN/TAA caused dramatic increases in inflammatory markers IL-6 and NLRP3 mRNA expression (850.93% (3.6 ± 5.779 vs. 0.33 ± 0.577, mRNA relative expression) and 732.14% (3.28 ± 5.229 vs. 0.33 ± 0.577, mRNA relative expression), respectively) compared to the control. HD-Cana treatment significantly reduced both IL-6 and NLRP3 expression (58.8% (1.47 ± 2.386 vs. 3.6 ± 5.779, mRNA relative expression) and 56.2% (1.43 ± 2.302 vs. 3.28 ± 5.229, mRNA relative expression) improvement from DEN/TAA, respectively), while LD-Cana showed moderate improvement (28.8% (2.61 ± 4.07 vs. 3.6 ± 5.779, mRNA relative expression) and 36.7% (2.3 ± 3.116 vs. 3.28 ± 5.229, mRNA relative expression). Neither dose completely normalized these inflammatory markers to control levels, though HD-Cana showed superior anti-inflammatory effects compared to LD-Cana.

### Histopathological findings

**Fig. 8:**
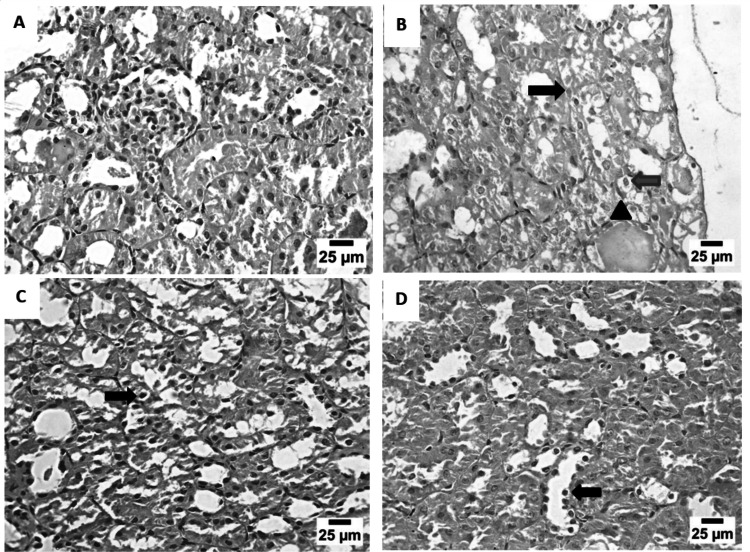
Photomicrographs of kidneys from different experimental groups stained with H&E. **A.** photomicrograph of control group showed no marked pathological changes in epithelium of renal tubules and glomeruli, **B.** photomicrograph of DEN/TAA showed eosinophilic solid and cystic renal cell carcinoma (black arrow) with sever vacuolar degeneration in epithelium of renal tubules (blue arrow), in addition to formation of renal cast (arrow head), **C.** photomicrograph of canagliflozin (10 mg/kg) group showed necrobiotic changes (including nuclear pyknosis and vacuolar degeneration) in some renal tubules (arrow), **D.** photomicrograph of canagliflozin (20 mg/kg) group showed nuclear pyknosis in some renal tubules (arrow)

Kidneys from the negative group showed normal renal parenchyma with normal renal glomeruli and renal tubules (Fig. 8A). In contrast, DEN/TAA group revealed eosinophilic solid and cystic renal cell carcinoma with sever vacuolar degeneration in epithelium of renal tubules, in addition to formation of renal cast (Fig. 8B). Kidney sections from canagliflozin (10 mg/kg) group showed including nuclear pyknosis and vacuolar degeneration in some renal tubules (Fig. 8C), while canagliflozin (20 mg/kg) group showed nuclear pyknosis in some renal tubules (Fig. 8D).

### Immunohistochemical staining

**Fig. 9:**
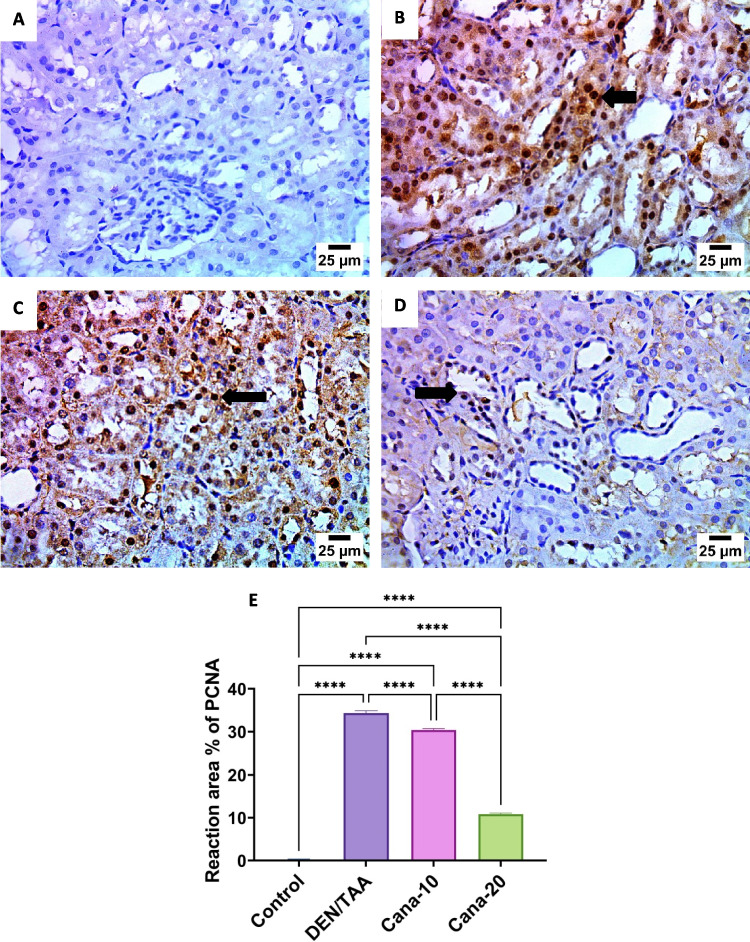
Expression of proliferating cell nuclear antigen (PCNA) in kidney tissues from different experimental groups (IHC-Peroxidase-DAB). **A.** photomicrograph of control group showed negative expression for PCNA in epithelium of renal tubules, **B.** photomicrograph of DEN/TAA group showed severe positive expression for PCNA in epithelium of renal tubules (arrow), **C.** photomicrograph of canagliflozin (10 mg/kg) group showed severe positive expression for PCNA in epithelium of renal tubules (arrow), **D.** photomicrograph of canagliflozin (20 mg/kg) group showed mild positive expression for PCNA in epithelium of renal tubules (arrow), **E.** PCNA expression in kidney tissues statistically significant at * *P* ≤ 0.05, ** at *P* ≤ 0.01, *** at *P* ≤ 0.001, and **** at *P* ≤ 0.0001

As represented in Fig. 9, control group showed negative expression for PCNA in epithelium of renal tubules. However, DEN/TAA group showed severe positive expression for PCNA in epithelium of renal tubules. On the other hand, canagliflozin (10 mg/kg) group showed severe positive expression for PCNA in epithelium of renal tubules, while canagliflozin (20 mg/kg) group showed mild positive expression for PCNA in epithelium of renal tubules.

**Fig. 10:**
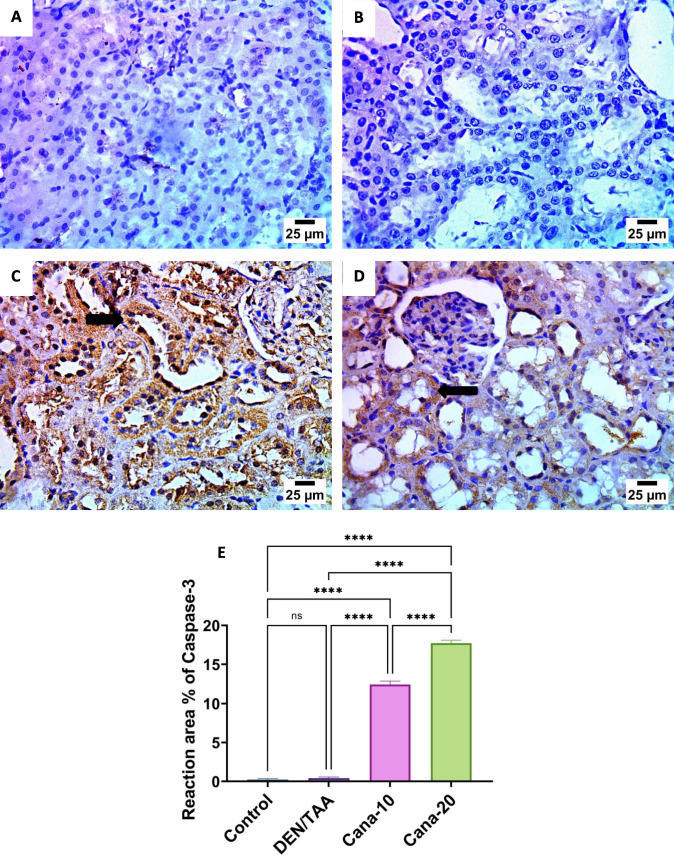
Expression of caspase-3 in kidney tissues from different experimental groups (IHC-Peroxidase-DAB). **A.** photomicrograph of control group showed negative expression for caspase- 3 in epithelium of renal tubules, **B.** photomicrograph of DEN/TAA group showed negative expression for caspase-3 in epithelium of renal tubules (arrow), **C.** photomicrograph of canagliflozin (10 mg/kg) group showed mild positive expression for caspase-3 in epithelium of renal tubules (arrow), **D.** photomicrograph of canagliflozin (20 mg/kg) group showed high positive expression for caspase-3 in epithelium of renal tubules, **E.** Caspase-3 expression in kidney tissues statistically significant at * *P* ≤ 0.05, ** at *P* ≤ 0.01, *** at *P* ≤ 0.001, and **** at *P* ≤ 0.0001

Figure 10 is showing the expression of caspase-3 in kidney tissues from different experimental groups. The control group showed negative expression for caspase-3 in epithelium of renal tubules. DEN/TAA group showed severe positive expression for caspase-3 in epithelium of renal tubules. Moreover, canagliflozin (10 mg/kg) group showed severe positive expression for caspase-3 in epithelium of renal tubules, contrariwise, canagliflozin (20 mg/kg) group revealed negative expression for caspase-3 in epithelium of renal tubules.

## Discussion

Owing to the role of the kidneys in filtering blood and concentrating xenobiotics, renal tissues are particularly susceptible to the oxidative and inflammatory damage induced by DEN and TAA. As well, the nephrotoxic potential of DEN and TAA arises from their capacity to generate reactive metabolites and oxidative stress, making the kidneys a primary target of injury in this model (Singh et al. 2021; Radwan et al. 2023). Experimental animals develop preneoplastic lesions in several organs due to DEN, a well-known carcinogenic N-nitroso compound (Mansour et al. 2019; Ramadan et al. 2020). When cytochrome P450 breaks down DEN, the body releases highly reactive free radicals that initiate “the lipid peroxidation process” and oxidatively damage DNA and proteins (Aiad et al. 2014). The administration of DEN in conjunction with TAA appears to induce carcinogenesis (Kimura et al. 2013). TAA is bioactivated by cytochrome P450 enzymes, which produce “TAASO and TAASO2”, hazardous substances that increase the generation of reactive oxygen species (ROS), lipid peroxidation, cytotoxicity, mitochondrial damage, and glutathione depletion (Rekha et al. 2008; Ramadan et al. 2018). While the DEN/TAA combination is indeed classically utilized for hepatocarcinogenesis models, several studies have demonstrated its capacity to induce multi-organ toxicity, including renal injury characterized by oxidative stress, inflammation, and tubular damage. This supports its relevance in modeling renal carcinogenesis under prolonged exposure conditions (Zhang and Xu 2024).

According to the current investigation results, DEN/TAA caused kidney injury in rats, which was demonstrated by elevated levels of blood indicators like creatinine and urea. Additionally, increased creatinine indicates renal impairment and reflects the glomerular function (Perrone et al. 1992). According to earlier research (Aly et al. 2017), rats exposed to DEN exhibited renal damage and a marked rise in circulating kidney function indicators, which confirmed our findings. By comparing to the rats that were given DEN/TAA combination, the treated groups given canagliflozin successfully improved renal functioning involving the levels of “urea and creatinine”. Similar findings (Park et al. 2022; Mourad et al. 2023) showed that canagliflozin improved renal functioning by lowering serum urea and creatinine levels. Histological examination of the renal tissue exposed to DEN/TAA verified these biochemical alterations. canagliflozin therapy, however, reversed these pathological changes.

Oxidative stress is a mismatch between ROS generation and the biological system's ability to counteract ROS's deleterious effects (Udensi and Tchounwou 2014). Excessive ROS can also weaken the antioxidant defense system, causing damage to proteins, lipids, and DNA (Kumar et al. 2008). Several renal disorders are primarily caused by oxidative stress, which also promotes the development of kidney cancer (Krata et al. 2018). In the present investigation, DEN/TAA injections led to a considerable increase in MDA level and a decrease in GSH content and SOD activity, suggesting a strong redox reaction inside the cell. These findings were corroborated by earlier research showed that DEN/TAA injections decreased renal GSH content and SOD activity while raising renal MDA levels (Iqbal et al. 2021; Elbaset et al. 2023). On the other hand, canagliflozin therapy dramatically reduced the high MDA level, indicating that canagliflozin treatment is successful in squelching free radicals. The upregulation of all renal antioxidant parameters further indicated the reno-protective benefits of canagliflozin treatment against DEN/TAA-induced oxidation stress. Du et al.’s work (Du et al. 2022) revealed similar results, indicating that canagliflozin lowers cardiac oxidative stress in diabetic cardiomyopathy-affected mice.

A key player in cell metabolism is AMP-activated protein kinase (AMPK) (Garcia and Shaw 2017). Stress triggers the activation of serine-threonine protein kinase, which is essential for preserving the equilibrium of cell energy (Hardie et al. 2016). Additionally, AMPK has been suggested as a crucial tumor suppressor protein in earlier researches (Luo et al. 2010; Yue et al. 2014). Activated AMPK inhibits mTOR complex 1 (mTORC1) (Inoki et al. 2003), induces autophagy (Kim and He 2013), activates p53 (Jones et al. 2005), and degrades a number of important oncogenic proteins (He et al. 2013) in order to achieve its anti-cancer effects. Additionally, the Warburg effect is inhibited by AMPK, which impacts oxidative phosphorylation (Cantó et al. 2009). A variety of anti-cancer medications have also been demonstrated to trigger AMPK-dependent cell death pathways (Chen et al. 2013; Lu et al. 2016). Therefore, one effective method of preventing cancer cells is AMPK activation. In line with the previously described function of AMPK in kidney cancer treatment, our results demonstrated that DEN/TAA injections markedly reduced AMPK level. In parallel, a study conducted by Wang et al. (Wang et al. 2021) showed that AMPK were decreased in RCC. Meanwhile, canagliflozin treatment reversed and returned AMPK to normal levels. In support of our findings, Packer’s study (Packer 2020) showed that in addition to inducing AMPK, SGLT2 inhibitors have been demonstrated to promote autophagy, which reduces cellular stress and glomerular and tubular damage. Wang et al. found that canagliflozin treatment, which blocks SGLT2, increased AMPK activation in thyroid cancer cells, hence reducing the growth of thyroid cancer cells *in vitro* and *in vivo* (Wang et al. 2022). According to these findings, canagliflozin showed anticancer properties via activating AMPK.

Most cancers are caused or worsened by inflammation, which lends credence to the idea that inflammation is a key factor in the development of carcinogenesis (Murata 2018). It is well known that inflammasomes, particularly NLRP3, cause carcinogenesis both *in vitro* and *in vivo* (Fan et al. 2014). RCC has been shown to have significant expression of NLRP3 (Mulay 2019). In addition to NLRP3, prior research has demonstrated that STAT3 signaling is crucial for the development of kidney malignancies, and higher STAT3 activation has been linked to the advancement of pathological stages and a lower overall survival rate (Horiguchi et al. 2002a, b). Because activated STAT3 hinders apoptosis and increases angiogenesis, proliferation, invasiveness, and immune evasion, it encourages the growth of tumors (Li et al. 2013). Aberrant STAT3 activation performs a serious role in the advancement of several cancer types (Yu and Jove 2004). STAT3 is constitutively active in human RCC and serves as an independent predictor (Horiguchi et al. 2002a, b). IL-6 is among the cytokines the inflammasome released from necrotic cells into the tumor microenvironment (Hanahan and Weinberg 2011). Consistent with these facts, our results demonstrated that DEN/TAA injections markedly increased IL-6 and NLRP3 expressions followed by activation of STAT3 levels. Several studies showed that the NLRP3 and IL-6/STAT3 pathways were increased after DEN/TAA injection (Park et al. 2010; Wang et al. 2015; Dwivedi and Jena 2020). Our data demonstrate that canagliflozin significantly downregulated renal expression of NLRP3, IL-6, and STAT3, suggesting a suppression of key pro-inflammatory and pro-oncogenic signaling pathways implicated in renal tumorigenesis, including the NLRP3/IL-6/STAT3 axis.

Nrf2 regulates and encodes antioxidant proteins by interacting with the antioxidant-responsive element (ARE) (Elbaset et al. 2024). Nrf2 normally binds with the Kelch-like ECH-associated protein-1 (Keap1) in the cytoplasm (Raslan et al. 2021). Upon exposure to DEN/TAA free radicals, Nrf2 is liberated from Keap1 and moves into the nucleus, triggering the antioxidant genes (Rotblat et al. 2012; Abdel-Rahman et al. 2024). Significantly, prior research has demonstrated the crucial function of the NRF2/KEAP1 signaling pathway in carcinogenesis and the relationship between redox and metabolism in cancer (Hayes and Dinkova-Kostova 2014), providing additional evidence that Nrf2 acts as an oncogene to encourage tumor cell motility and invasion, potentially by boosting the tumor’s resistance to oxidative stress. It is increasingly evident that the NRF2/KEAP1 pathway contributes significantly to the metabolic reprogramming of cancer cells by means of a transcriptional program that promotes cancer cell proliferation and malignant development. Furthermore, Nrf2 promotes intermediate metabolism through glutaminolysis (Romero et al. 2017), which leads to an imbalance in metabolic activities, including nucleotide (Mitsuishi et al. 2012) and amino acid biosynthesis (DeNicola et al. 2015). The findings revealed that Nrf2 plays an oncogenic role in the formation of renal cancer since the group with renal cancer had elevated Nrf2 levels, which were reversed to normal levels in the groups receiving canagliflozin treatment.

Dysregulation of apoptosis (programmed cell death) is believed to contribute to cancer progression (Wyllie 1997). Apoptotic cell death is initiated and carried out by members of the caspase family of cellular proteases. An important protease in the apoptotic process is caspase 3 (McIlwain et al. 2013). Immune expression of the cell proliferation marker PCNA and caspase-3 showed that the renal cancer group had significantly higher expression of PCNA and significantly lower expression of caspase-3. Findings of the current study were reinforced by the Dwivedi study, which showed that the group exposed to DEN + TAA had considerably higher PCNA expression (Dwivedi and Jena 2020). The expression of caspase-3 was upregulated, while PCNA was downregulated upon canagliflozin therapy at the two examined dose levels. Comparable results were reported in a study by Jojima et al. (Jojima et al. 2019) demonstrating that canagliflozin triggered caspase-3 to cause HepG2 cells to undergo apoptosis.

While the current findings provide important insight into the protective effects of canagliflozin in a chemically induced renal carcinogenesis model, some limitations should be acknowledged. First, while total RNA purity was confirmed using A260/A280 ratios within the accepted range (1.8–2.0), no-template controls (NTCs) were not included in each real-time PCR run. This omission is now noted as a methodological limitation and will be addressed in future studies for enhanced assay specificity. Secondly, although image analysis (using ImageJ software) was performed to quantify immunohistochemistry, some reliance on visual scoring and manual field selection may introduce observer bias. Blinded scoring and automated batch analysis would enhance objectivity.

## Conclusion and prospective

Findings of the current study demonstrate that canagliflozin reduced renal oxidative stress, as evidenced by decreased MDA levels and increased antioxidant capacity. It also improved kidney function markers (e.g., serum creatinine and urea) and promoted tumor apoptosis, as indicated by elevated caspase-3 expression. Mechanistically, canagliflozin suppressed components of the NLRP3/IL-6/STAT3 and Nrf2 pathways, suggesting attenuation of pro-inflammatory and pro-oncogenic signaling. Concurrently, AMPK activation may have contributed to canagliflozin’s protective and antitumor effects. To our knowledge, this is the first study exploring canagliflozin’s impact on experimental renal carcinogenesis. These findings provide a basis for future studies investigating its therapeutic potential and molecular mechanisms in RCC management.

## Data Availability

Raw data are available upon request.
